# Estimating Energy Expenditure from Heart Rate in Older Adults: A Case for Calibration

**DOI:** 10.1371/journal.pone.0093520

**Published:** 2014-04-30

**Authors:** Jennifer A. Schrack, Vadim Zipunnikov, Jeff Goldsmith, Karen Bandeen-Roche, Ciprian M. Crainiceanu, Luigi Ferrucci

**Affiliations:** 1 Longitudinal Studies Section, Translational Gerontology Branch, National Institute on Aging, National Institutes of Health, Baltimore, Maryland, United States of America; 2 Department of Epidemiology, Johns Hopkins Bloomberg School of Public Health, Baltimore, Maryland, United States of America; 3 Department of Biostatistics, Johns Hopkins Bloomberg School of Public Health, Baltimore, Maryland, United States of America; 4 Center on Aging and Health, Johns Hopkins Medical Institutions, Baltimore, Maryland, United States of America; Université de Montréal, Canada

## Abstract

**Background:**

Accurate measurement of free-living energy expenditure is vital to understanding changes in energy metabolism with aging. The efficacy of heart rate as a surrogate for energy expenditure is rooted in the assumption of a linear function between heart rate and energy expenditure, but its validity and reliability in older adults remains unclear.

**Objective:**

To assess the validity and reliability of the linear function between heart rate and energy expenditure in older adults using different levels of calibration.

**Design:**

Heart rate and energy expenditure were assessed across five levels of exertion in 290 adults participating in the Baltimore Longitudinal Study of Aging. Correlation and random effects regression analyses assessed the linearity of the relationship between heart rate and energy expenditure and cross-validation models assessed predictive performance.

**Results:**

Heart rate and energy expenditure were highly correlated (r = 0.98) and linear regardless of age or sex. Intra-person variability was low but inter-person variability was high, with substantial heterogeneity of the random intercept (s.d. = 0.372) despite similar slopes. Cross-validation models indicated individual calibration data substantially improves accuracy predictions of energy expenditure from heart rate, reducing the potential for considerable measurement bias. Although using five calibration measures provided the greatest reduction in the standard deviation of prediction errors (1.08 kcals/min), substantial improvement was also noted with two (0.75 kcals/min).

**Conclusion:**

These findings indicate standard regression equations may be used to make population-level inferences when estimating energy expenditure from heart rate in older adults but caution should be exercised when making inferences at the individual level without proper calibration.

## Introduction

Physical activity energy expenditure is a modifiable risk factor for multiple chronic diseases. Previous research has demonstrated that higher levels of activity energy expenditure are associated with better health outcomes in older adults [1], [2]. Accurate measurement of free-living energy expenditure is therefore vital to further understanding and quantifying changes in energy metabolism with aging and their effects on activity, disability, and disease in population-based studies of older adults.

Questionnaire-based methods provide valid estimates of easily-remembered episodes of moderate-to-vigorous activity, but their ability to accurately estimate total free-living energy expenditure across a wide range of intensity is controversial [3]–[5]. Physical activity records may provide an acceptable estimate of energy expenditure but require good subject compliance and provision of careful instructions [6] that may not be feasible in older study populations. Thus, a valid and precise assessment of free-living energy expenditure requires 24-hour monitoring in free-living conditions. The doubly-labeled water method allows quantification of energy expenditure over a prolonged period and is generally believed to be the gold standard [7], but its cost makes it unfeasible in medium-to-large epidemiological studies and it cannot quantify subcomponents of energy expenditure such as intensity, duration, and frequency.

Heart rate monitoring has been postulated as an available objective method for monitoring free-living activity in large study populations, but its validity and reliability remain unclear [3], [8]. The efficacy of heart rate as a surrogate for energy expenditure is rooted in the assumption that - in most individuals - there is a valid linear function relating heart rate and energy expenditure [9], [10]. Previous efforts to quantify the relationship have primarily focused on modeling the heart rate – energy expenditure trajectory in small to medium sized studies (N ranging from 12–89) of mostly younger individuals who are generally fitter than the average population [10]–[17], and have not fully addressed the relationship in an older population with a more limited heart rate range. Further, because of limitations in sample size and participant age these studies did not establish whether standard equations may be widely applicable to individuals of different ages or whether greater age increases the heterogeneity of the energy expenditure estimates. Although individual calibration has been shown to reduce error in younger populations, the magnitude of the reduction is unclear [16], [17]. Ideally, to enhance usability across studies, calibration methods should be as simple and minimal as possible while enhancing accuracy and applicability to large population-based studies. Quantifying the amount of precision gained with each level of individual calibration will help establish a calibration hierarchy for estimating energy expenditure from heart rate in older adults.

The primary objectives of this study were to: (1) examine the relationship between heart rate and energy expenditure across five distinct levels of exertion in a large population of older adults, and (2) to assess the error size and potential age-bias introduced by estimating energy expenditure from heart rate at the individual and population levels with and without calibration data. Limitations in sample size, physical function, and health status have thus far precluded examination of these variables in a large sample of older adults, limiting the relevance of heart rate as an objective measure in the older population. Evaluating the linearity and heterogeneity of this relationship across multiple tests in a large sample of older adults will strengthen its validity and enhance the ability to monitor free-living energy expenditure in research and clinical settings.

## Materials and Methods

The Baltimore Longitudinal Study of Aging (BLSA) is a study of normative human aging, established in 1958 and conducted by the National Institute on Aging Intramural Research Program. A general description of the sample and enrollment procedures and criteria has been previously reported [18]. Briefly, the BLSA is a continuously enrolled cohort with some targeted recruitment (e.g., women, racial minorities) over its history. All participants are community volunteers who must pass a comprehensive health and functional screening evaluation and be free of all major chronic conditions and cognitive and functional impairment at the time of enrollment. Once enrolled, participants are followed for life and undergo extensive testing every one to four years depending on age. The current clinical enrollment of the BLSA is 1133 individuals (217 participants are seen in-home) with an age-range of 28–97. Although the age range of the BLSA is broad, only 24% of participants are younger than age 60, which allows observation of aging across the lifespan, with an emphasis on older ages.

The sample for the current study consists of men and women who underwent a physical examination, health history, and comprehensive energy expenditure testing during their visit (n = 375) between July 2007 and August 2011. Of these participants, 17 were excluded for use of cardiac medications, such as beta-blockers, which may induce a blunted heart rate/energy expenditure response. The remaining tests were examined for accuracy and 68 participants were excluded for one or more questionable or invalid tests resulting from: equipment calibration error, missing heart rate data, or invalid test length. The final sample includes 290 individuals with five high quality energy expenditure tests across the range from resting to maximal exertion. Trained and certified technicians administered all assessments following standardized protocols. The Internal Review Board of the Medstar Research Institute approved the study protocol and all participants provided written informed consent.

Participants were admitted to the Clinical Research Branch unit of the National Institute on Aging for three days of testing. Height and weight were assessed in light clothing using a stadiometer and calibrated scale, respectively. Date of birth (age) was derived from a health history interview conducted by trained technicians. Energy expenditure was assessed at rest and at four intensities: slow-walking, customary walking, peak sustained walking, and maximal exertion.

### Heart Rate and Energy Expenditure Assessment

Heart rate and energy expenditure were assessed simultaneously using either: (1) a Polar Heart Rate Monitor (Polar, USA) and a Cosmed k4b^2^ portable metabolic analyzer (Cosmed, Rome, Italy), or (2) a 12-lead EKG and a Medgraphics D-series metabolic cart (Medical Graphics Corp., St. Paul, MN). The BLSA utilizes two different metabolic analyzers to allow assessment of energy expenditure during both treadmill and overground walking. Previous validation testing between analyzers shows consistent results [19]. Prior to testing, both analyzers were calibrated using a 3.0 liter flow syringe and gases of known concentrations. Both analyzers collect gas-exchange data on a breath-by-breath basis averaged over 30 second intervals to reduce variability. The resting, customary walking, and peak sustained walking tests, utilize the Cosmed analyzer and the slow-walking and maximal exertion tests utilize the Medgraphics analyzer. Energy expenditure was calculated as the average volume of oxygen consumed per kilogram of body weight (VO_2_ ml/kg/min) for each test.

Resting heart rate and energy expenditure were assessed for 16 minutes first thing in the morning after an overnight stay in a quiet, thermo-neutral environment, prior to eating or drinking. The average heart rate and energy expenditure from minutes 5.5–15.5 were used in the analysis.

Slow-walking heart rate and energy expenditure were assessed for five minutes on a motorized treadmill at a speed of 0.67 m/s, 0% grade. Average heart rate and energy expenditure were derived from the final three minutes of testing.

Customary walking heart rate and energy expenditure and peak-sustained heart rate and energy expenditure were assessed during the “long-distance corridor walk” (LDCW), a two-part validated measure of cardiorespiratory fitness in older adults [20], [21]. The tests were performed sequentially on a 20-meter course in an uncarpeted corridor while wearing the Cosmed analyzer.

#### Customary walking test

Participants stood behind a taped starting line and were instructed to walk at their “usual comfortable pace” around the course in a continuous loop until directed to stop. After a command of “Go,” timing was initiated with the first foot-fall over the starting line and stopped after 2.5 minutes of customary walking. Average heart rate and energy expenditure during customary walking were derived from the final minute of testing.

#### Peak sustained walking test

Peak-sustained walking heart rate and energy expenditure were assessed immediately following the customary walking test. Participants were escorted back to the starting line and instructed to walk “as fast as possible, at a pace you can sustain for 400 meters.” Timing was initiated with the first foot-fall over the starting line and stopped after 400 meters of peak sustained walking. Standardized encouragement was given each lap along with the number of laps remaining. Average heart rate and energy expenditure during peak sustained walking were derived by removing the first 1.5 minutes of the 400 m walk and averaging the remaining minutes.

Maximal exertion testing was performed on a motorized treadmill. Oxygen consumption was measured continuously during a modified Balke protocol. For the majority of participants, speed was held constant at 3.0 mph for women and 3.5 mph for men while the grade of the treadmill progressively increased 3% every 2 minutes until voluntary exhaustion. In participants of higher fitness, treadmill speed was increased by 0.5 mph 1 to 3 times during the test. Oxygen consumption was calculated in 30 second intervals and the highest value was labeled maximal exertion (VO_2_ max). All participants met or exceeded a respiratory exchange ratio of 1.05.

### Statistical Analysis

**Figure 1:**
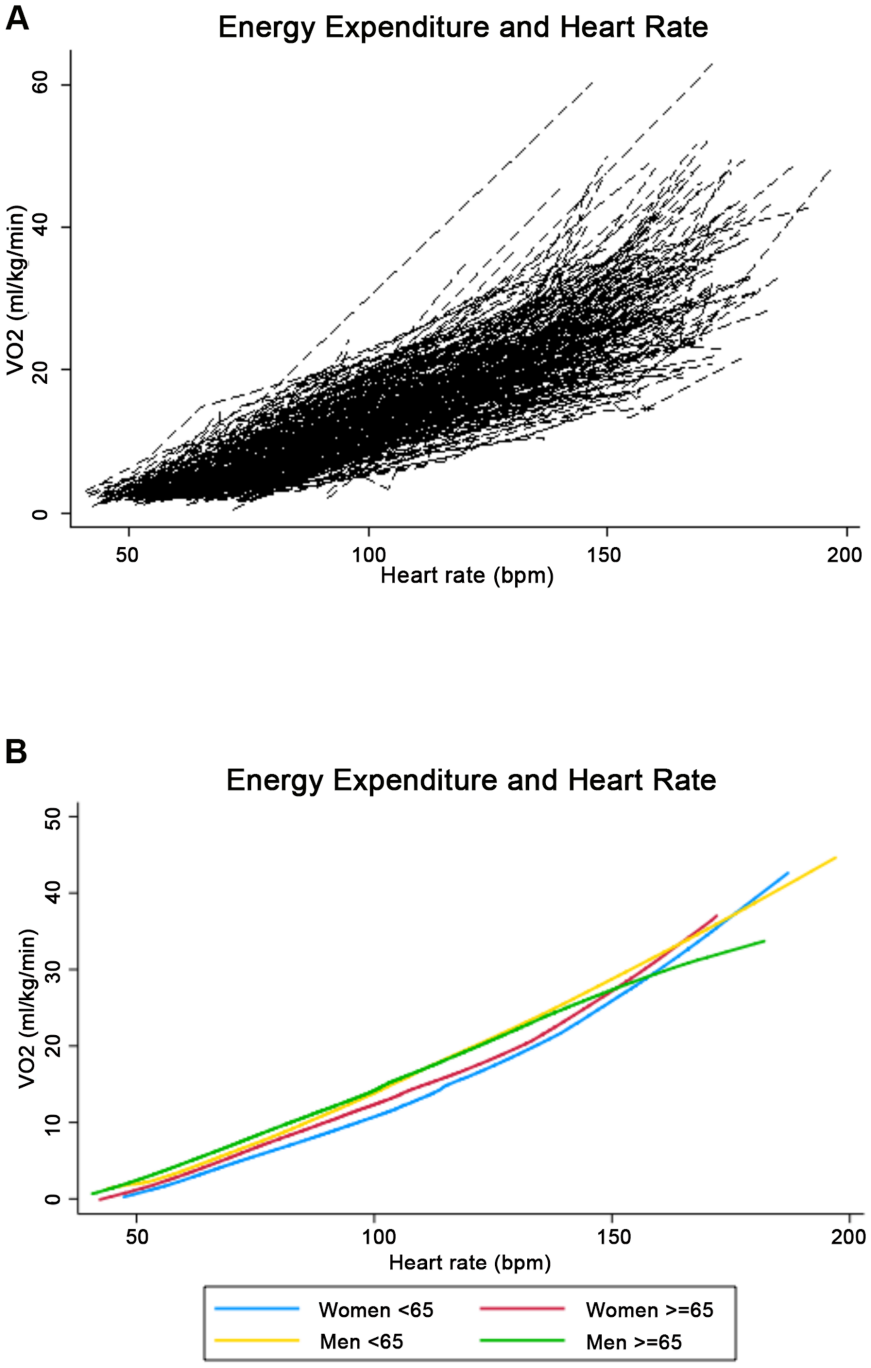
a shows a spaghetti plot (N = 290) of the relationship between heart rate (bpm) and energy expenditure (ml/kg/min). b shows an age and sex stratified LOWESS of the relationship between heart rate (bpm) and energy expenditure (ml/kg/min).

Unadjusted relationships between heart rate and VO_2_ (ml/kg/min) were explored with a spaghetti plot and summarized by locally weighted regression smoothers (Figures 1a and 1b). Based on the appearance of these Figures, two models were generated to determine optimal fit: a stratified model (Model 1) and a group equation model (Model 2).

In Model 1, four sex and age strata were created using group indicator variables G1, G2, G3, and G4 to represent men younger than 65, men 65 and older, women younger than 65, and women 65 and older, respectively. Using G1 as the reference group, the following random effects model was generated with a random intercept and random slope for each subject:
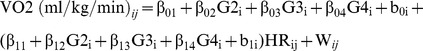
Where β_01_ is the intercept for men less than 65, β_01_+β_02_ is the intercept for men 65 and older, β_01_+β_03_ is the intercept for women less than 65, and β_01_+β_04_ is the intercept for women 65 and older. The corresponding heart rate slope for each stratum is β_11_, β_11_+β_12_, β_11_+β_13_, and β_11_+β_14_, respectively. In Model 2, age was treated as a continuous variable while adjusting for sex and the interaction between age and heart rate
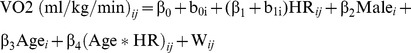
Where β_0_ is the mean intercept and β_1_ is the mean slope. In both models, the subject-specific random intercept (b_0i_) and random slope (b_1i_) were modeled jointly as zero mean bivariate normal random variables with unconstrained covariance matrix, or more simply variances of random slope and random intercepts as well as their correlation coefficient were estimated individually from the data, and *W_ij_*'s were modeled as normal random variables with zero mean and a constant variance across subjects. Height and the interaction terms for sex and height and sex and age were not significant and excluded from both models.

### Cross Validation

To compare the predictive performance of Models 1 and 2 the dataset was randomly divided into training and validation sets using 80% of the data as training and 20% as validation. For Model 1, randomization was performed at the subject-level after stratifying into the four sex/age categories. Using the training data, Models 1 and 2 and parameter estimates from these models were subsequently used to predict VO_2_ ml/kg/min for subjects in the validation dataset. The predictive ability of each model was assessed with different levels of calibration and subject specific effects: (1) no calibration data, subject specific effects set to zero; (2) resting heart rate and energy expenditure; (3) resting heart rate and energy expenditure and peak heart rate and energy expenditure; (4) all five levels of heart rate and energy expenditure, from resting to maximal.

Analyses were performed using Stata MP, version 10 (Statacorp, College Station, TX) and R, version 2.14.1 (R Foundation for Statistical Computing, Vienna, Austria). P-values of <.05 were considered significant.

## Results

Participant characteristics including mean heart rate and energy expenditure values are shown in Table 1. The overall age range was 32–90 (mean 67.6±11.5) years. There were more men in the study sample (59%) than women (41%) as fewer female participants completed maximal treadmill testing to the required threshold. The study encompassed a wide age range, resulting in considerable variation in the range of energy expenditure values. Despite this variation, the overall intra-person correlation (r) was 0.98 with a range of 0.93–0.99. The strength of this association is portrayed in Figures 1a and 1b. With the exception of a few outliers (Figure 1a), the relationship between heart rate and oxygen consumption is remarkably linear both within subjects and at the population level. As depicted in Figure 1b, women tended to have slightly lower oxygen consumption (ml/kg/min) at a given heart rate until approaching peak or maximal capacity at which time the distinction among the strata becomes unclear.

**Table 1:** Participant characteristics.

N = 290	Mean	± SD/%
Age, years	67.6	11.5
Male sex, no.	172	59.3
Weight, kg	79.2	14.4
Height, cm	171.0	9.1
Body mass index, kg/m^2^	27.0	4.2
Resting heart rate, bpm	61.2	9.7
Slow-walking heart rate, bpm	85.7	12.9
Customary walking heart rate, bpm	97.8	15.7
Peak sustained walking heart rate, bpm	120.4	19.8
Maximal exertion heart rate, bpm	148.6	21.0
Resting energy expenditure, ml/kg/min	2.7	0.7
Slow walking energy expenditure, ml/kg/min	8.6	1.6
Customary walking energy expenditure, ml/kg/min	12.9	2.7
Peak sustained walking energy expenditure, ml/kg/min	18.5	4.8
Maximal energy expenditure, ml/kg/min	29.1	9.3

kg, kilograms; cm, centimeters; kg/m^2^, kilograms per meter squared; bpm, beats per minute; ml/kg/min, milliliters per kilogram per minute.

The results of Model 1 are shown in Table 2. The intercepts and heart rate slopes were −15.39 ml/kg/min and 0.28 for men less than 65, −17.59 ml/kg/min and 0.29 for women less than 65, −14.96 ml/kg/min and 0.30 men 65 and older, and −17.06 ml/kg/min and 0.29 for women 65 and older. The standard errors of the random intercept and slope were 0.374 and 0.004, respectively, indicating differences in sex-and age-specific intercepts, but substantially no difference in heart rate slopes across strata once subject specific random effects are taken into account.

**Table 2:** Stratified Model 1 Parameter Estimates.

Parameter	Coefficient	p-value/std. error
Men <65 Intercept (β_01_)	−15.392	<.001
Men ≥65 Intercept (β_01_+β_02_)	−14.960	.483
Women <65 Intercept (β_01_+β_03_)	−17.591	.001
Women ≥65 Intercept (β_01_+β_04_)	−17.055	.018
Men <65 HR slope (β_11_)	.296	<.001
Men ≥65 HR slope (β_11_+β_12_)	.279	.139
Women <65 HR slope (β_11_+β_13_)	.297	.936
Women ≥65 Intercept (β_11_+β_14_)	.285	.397
sd(b_0i_)	4.007	(.374)*
sd(b_1i_)	0.063	(.004)*
corr(b_0i_, b_1i_)	−0.875	(.022)*
sd(W_ij_)	2.785	(.062)*

HR, Heart rate.

*std. errors are reported in parenthesis.

The results of Model 2, the group random effects model, are shown in Table 3. Heart rate remained a significant independent predictor of energy expenditure after adjusting for sex, age, and the interaction between age and heart rate. The standard error of the random intercept (0.372), and random slope (0.004) were virtually unchanged from the stratified model indicating similar model fit. The mean intercept of the study population was −23.05 ml/kg/min and the mean heart rate slope was 0.34. Table 4 provides a detailed comparison of observed versus estimated energy expenditures (using Model 2) for several representative age-sex categories. Note that age stratification of Model 1 is replaced with the statistically significant heart rate and age interaction in Model 2.

**Table 3:** Model 2 Group Equation Parameter Estimates.

Parameter	Coefficient	p-value/std. error
Intercept	−23.046	<.001
HR	.339	<.001
Male	2.241	<.001
Age	.099	<.001
Age*HR	−.0007	.038
sd(b_0i_)	3.966	(0.372)*
sd(b_1i_)	0.062	(0.004)*
corr(b_0i_, b_1i_)	−0.874	(0.023)*
sd(W_ij_)	2.787	(0.062)*

HR, heart rate; Male, male sex; Age*HR, the interaction between age and heart rate.

*std. errors are reported in parenthesis.

**Table 4:** The average heart rate (bpm), observed energy expenditure (ml/kg/min), and estimated energy expenditure (ml/kg/min) of four representative age groups across five calibration levels (rest, slow-walking, customary walking, peak sustained walking, and maximal exertion), stratified by gender.

Age (years)		Rest			Slow Walking			Customary Walking			Peak Sustained Walking			Maximal exertion		
		HR	OEE	EEE	HR	OEE	EEE	HR	OEE	EEE	HR	OEE	EEE	HR	OEE	EEE
**51–60**	**M**	62.0	2.9	3.3	86.4	8.5	10.6	99.8	13.6	14.6	135.5	23.0	25.4	167.7	32.2	35.0
	**F**	65.2	2.4	2.1	90.8	8.3	9.7	104.6	12.5	13.9	130.9	18.5	21.8	159.1	27.8	30.2
**61–70**	**M**	59.2	2.8	3.1	81.1	8.4	9.4	95.4	13.4	13.7	116.7	19.3	19.9	147.2	26.8	28.9
	**F**	63.1	2.5	1.9	90.6	8.2	10.0	104.8	12.4	14.1	125.2	16.7	20.1	151.0	24.1	27.7
**71–80**	**M**	57.7	2.7	3.1	81.9	8.9	10.1	94.7	13.3	13.8	115.0	17.9	19.6	140.9	24.3	27.0
	**F**	62.2	2.6	2.2	92.8	8.9	11.0	104.1	12.9	14.2	121.7	16.5	19.2	143.0	22.6	25.4
**81–90**	**M**	58.9	2.7	4.0	83.9	9.3	11.0	93.8	12.9	13.8	108.3	16.8	17.9	135.2	23.4	25.4
	**F**	60.2	2.3	2.1	85.0	8.4	9.0	96.1	11.6	12.2	110.2	14.2	16.1	125.3	19.5	20.4

HR = Heart rate (bpm).

OEE = Observed energy expenditure (ml/kg/min).

EEE = Estimated energy expenditure (ml/kg/min).

**Figure 2:**
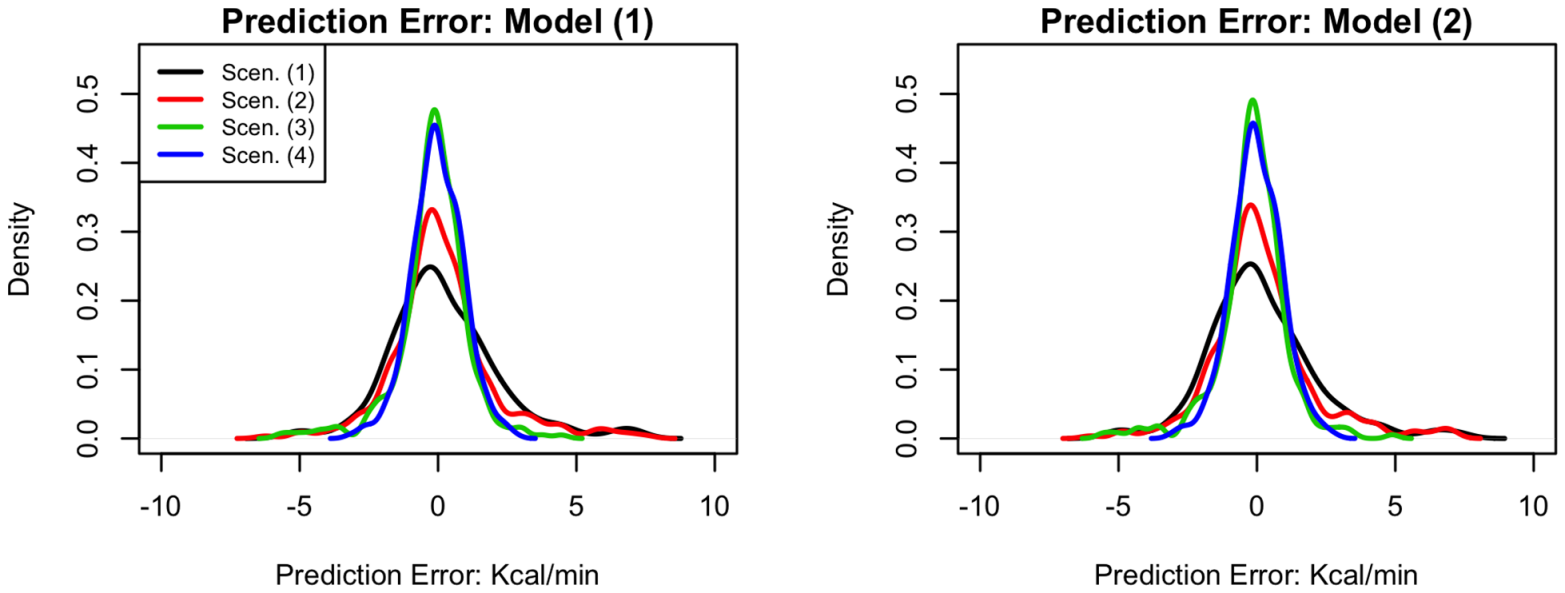
Figure 2 shows Kernel density estimates for the distribution of prediction errors for the four calibration data scenarios considered: (1) no calibration data, (2) resting heart rate and energy expenditure, (3) resting and peak heart rates and energy expenditures, and (4) all five levels of heart rate and energy expenditure. The left panel shows prediction errors computed under model (1), which uses four age/gender categories to predict energy expenditure, and the right panel prediction errors computed under model (2), which uses gender and age as continuous variables, and their interaction to predict energy expenditure.

**Figure 3:**
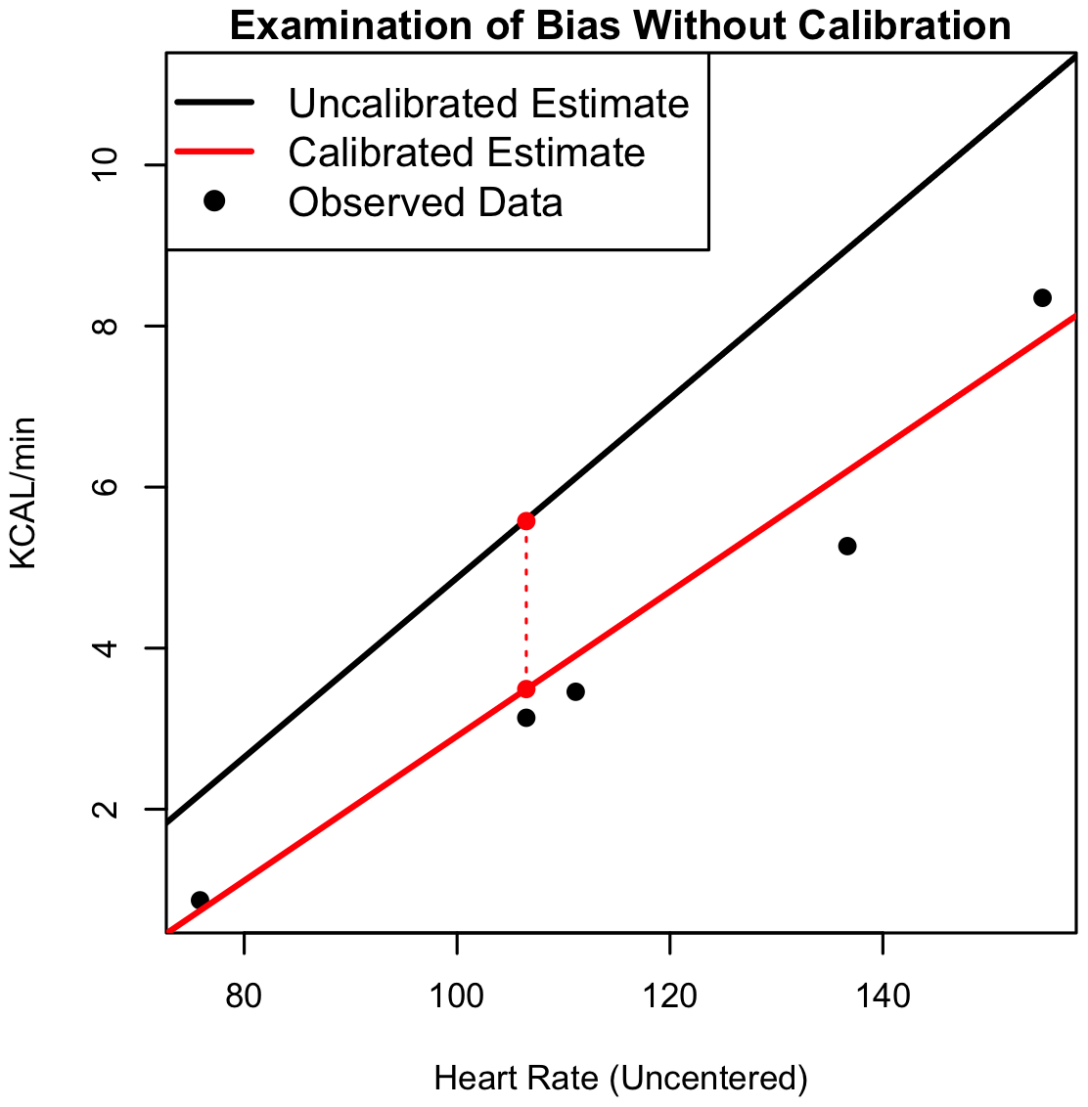
Figure 3 shows an Illustration of the difference between population-level and individual-level relationships between heart rate and energy expenditure. The solid black is the population equation, estimated from the subject's age and sex, omitting subject-specific random effects; the solid red line is the subject equation including the subject-level random-effects; and the vertical dashed red line shows the difference between the population-level and subject-level equations at the slow walking heart rate.

Figure 2 illustrates the results of the cross validation procedure. Energy expenditure was converted from VO_2_ ml/kg/min to kcals/min using a conversion factor of 5 kcals/L of O_2_ [22] to enhance interpretability. The left panel shows the distribution of prediction errors under Model (1) for each of the four calibration data scenarios: (1) no calibration data, (2) resting heart rate and energy expenditure, (3) resting and peak heart rate and energy expenditure, and (4) all five levels of heart rate and energy expenditure. Increasing the amount of calibration data improved the predictive ability of the models, as shown by the narrowing of the distributions and the decreasing standard deviation of prediction errors (2.03, 1.86, 1.26, and 0.94 kcals/min for each of the four calibration levels, respectively). The right panel of Figure 2 provides the distribution of prediction errors calculated under Model (2). The distribution of errors was similar for those calculated under Model (1), indicating the two models have similar predictive performance. Again, model performance also improved as the amount of calibration data increased (standard deviations of prediction errors were 2.01, 1.85, 1.26, and 0.93 kcals/min for the calibration scenarios). Finally, Figure 3 demonstrates the importance of including random effects when modeling energy expenditure in a specific individual. The solid black line illustrates the relationship between heart rate and energy expenditure (kcals/min) using the population equation (Model 2) with no calibration data (setting random effects to zero). The red line shows a subject-specific equation, which uses the individual's calibration data to estimate a subject-level intercept and slope. The difference between the two lines is generally between 1–2 kcals/min, which could lead to substantial error when estimating energy expenditure over the course of a day.

## Discussion

Objective and accurate measurement of free-living energy expenditure is imperative to understanding the best mechanisms for preventing disease and disability in an aging population. Heart rate monitoring provides an objective cost-effective method to monitor the intensity, duration, and frequency of daily activities using a physiological parameter that internally senses exercise intensity and cardiovascular adaptation to it. However, the ability to derive energy expenditure from heart rate depends on accurate calibration and evaluation of the relationship between heart rate and energetic demand. This study evaluated the heart rate response to energetic demand across five separate tests of exertion in a large population of older adults. The major findings indicate that the heart rate – energy expenditure trajectory is remarkably linear, regardless of age or sex, with low intra-person variability, and that energy expenditure may be reasonably estimated from heart rate at the population level. However, the possibility for substantial prediction error without calibration data may contribute to considerable inaccuracies when predicting daily energy expenditure at the individual level.

When interpreting the results, it is important to emphasize the distinction between population- and subject-level analyses. In both models, the random effects are a key source of variability, indicating that even after adjusting for major covariates a significant amount of inter-subject heterogeneity remains. The vertical distance between the lines of Figure 3 (dashed red line) represents the bias induced by using the population equation to estimate subject-level energy expenditure. Over time, this subject-specific bias may be a substantial source of error when estimating daily energy expenditure. In the data considered here, 67% of subjects have an estimated random intercept with absolute value higher than 0.35; over the course of twenty-four hours this could lead to over- or underestimating cumulative energy expenditure by 500 calories per day.

Importantly, it should be noted that population equations may be useful to summarize and compare average differences in energy expenditure among groups: setting the random intercept and random slope to zero for large populations will not greatly impact averages. However, because significant differences exist between subjects with similar characteristics, the use of population equations to estimate energy expenditures at the individual level in small laboratory or clinical settings may lead to significant errors. The primary source of variation appears to come from the heterogeneity of the intercept, or the relationship between resting heart rate and energy expenditure or “flex” heart rate. This is not surprising considering the wide variation in resting heart rate among individuals, even after controlling for age and sex, due to wide variability in cardiovascular fitness and is consistent with previous studies in smaller, younger populations [12], [13], [16], [23].

This study has several major strengths. (1) The size and age-range of the study sample allowed examination of the heart rate - energy expenditure trajectory across a large age range, with an emphasis on older adults, a previously under-assessed population. (2) The use of resting energy and four measures of exertion enhances the ability to estimate energy expenditure across levels of daily activity. Further, the inclusion of two mid-to-low exertional measures enhances accuracy near flex-heart rate, where most daily energy is expended [24]. (3) The use of random effects models to assess the key components of model variability. (4) Finally, the use of prediction models to estimate model accuracy with and without calibration data.

Although these methods lend strength to the equations generated, it is not feasible for many studies to quantify the heart rate - energy expenditure relationship across five levels of exertion and calibrate accordingly. However, results of the cross-validation indicate substantial improvements in accuracy may be obtained by adding a single calibration measure to the equation. Thus, obtaining one individual calibration measure of resting heart rate and oxygen consumption could be the best compromise between validity and feasibility.

All of the performance tests included in the analyses employed walking as the preferred method of exertion. Although this does not allow assessment of energy expenditure during other patterns of movement, the majority of daily activities require ambulation equivalent to walking and calibration procedures should reflect the most common activity of the population in question [14]. Moreover, walking was assessed at slow, customary, and peak paces to encompass the full range of demand of most daily activities.

The findings of high inter-person variability are consistent with previous research [3], [12], [16], [17], [25]. The current research builds on this work by exploring this phenomenon at the population level and suggesting this variability may be lessened with minimal calibration, making it an attractive option for objectively monitoring energy expenditure in large population studies. Although the potential for bias still exists even with individual calibration, it is substantially lessened, and provides the potential for more accurate assessment than subjective methods which have been found to overestimate energy expenditure by as much as 62% [26]. Further validation of these equations in other older adult populations and using doubly labeled water estimates of energy expenditure is warranted to verify these implications.
